# The Post-Roe Political Landscape Demands a Morality of Caution for Women’s Health

**DOI:** 10.2196/41417

**Published:** 2022-10-20

**Authors:** Sarah Goodday, Daniel Karlin, Christine Suver, Stephen Friend

**Affiliations:** 1 4YouandMe Seattle, WA United States; 2 Department of Psychiatry University of Oxford Oxford United Kingdom; 3 Department of Psychiatry Tufts University School of Medicine Boston, MA United States; 4 MindMed Inc New York, NY United States; 5 Sage Bionetworks Seatlle, WA United States

**Keywords:** women's health, reproductive health, wearable, abortion rights, confidentiality and privacy, Roe v. Wade, health policy, health research, reproductive information, privacy, women's rights, health rights, abortion, eHealth, digital health, mHealth, safety, ethic

## Abstract

The recent Supreme Court decision (ie, Dobbs v. Jackson Women’s Health Organization), revoking the constitutional right to abortion in the United States, has the potential to dramatically disrupt progress in women’s health research. The typical safeguards to ensure confidentiality and privacy of research participants in studies that collect certain types of personal health information may not hold against criminal investigations surrounding suspected pregnancy terminations. There are additional risks to participants in digital health research studies involving the use of wearable devices capable of tracking physiological measures, such as body temperature and heart rate, as these have shown promise for tracking conception and could be used to identify pregnancy termination signatures. There are strategies researchers can use to protect the safety of participants in health research who could get pregnant, while also maintaining integrity of research methods. The objective of this viewpoint is to discuss potential strategies to protect research participants’ privacy that include the minimization of nonessential sensitive personal health information and anonymization protocols in the event of miscarriage or termination of pregnancy. We invite others to join this discussion so as to not let the current political landscape impede progress in women’s health and reproductive research, while also protecting research participants.

## Introduction

The US Supreme Court’s decision involving the case Dobbs v. Jackson Women’s Health Organization (Dobbs v. Jackson ruling) in June 2022 to overturn Roe v. Wade, thereby dismissing the constitutional right to abortion [1] led to several US states taking rapid action to ban, restrict, and criminalize abortion. This decision will have significant negative impacts on maternal and child health, their economic welfare, well-being outcomes, and mortality in the United States, disproportionately impacting those from disadvantaged populations [2,3]. This decision will also dramatically disrupt progress in women’s health and reproductive research.

Numerous policy efforts have been developed to enhance the inclusion in health research of women and other people who can get pregnant, especially in light of the historical exclusion of this group, including many efforts by the National Institutes of Health [4-8]. The Dobbs v. Jackson ruling dispiritedly impacts this progress, where participants in health research who can get pregnant are now at an increased risk of having certain types of personal health information used against them by some states or other individuals. Until now, investigators were able to rely on maintaining confidentiality of their research participants through protections granted by federal or state statutes, for example, through Health Insurance Portability and Accountability Act (HIPAA) compliance, which applies restrictions on the use and disclosure of personal health information. Additional layers of protection could be added through safeguards, such as a Certificate of Confidentiality, that are issued pursuant to the 21st Century Cures Act’s amendments to the Public Health Services Act [9] and allows participants and investigators to refuse the disclosure of research data in the event of a federal, state, or local request; however, both HIPAA and the Certificate of Confidentiality have exclusions that are not universally applicable in research projects in the United States. Since the Dobbs v. Jackson ruling, these governance safeguards have been called into question, suggesting that they may not be sufficient to protect participants’ confidentiality in the face of a criminal investigation related to suspicion of abortion [10-12]. These recent developments have sparked discussion on additional mechanisms to protect patient privacy, especially in states that criminalize abortion and more so in those that work to restrict individuals’ ability to travel out of state to access abortion clinics. As of August 2022, a total of 12 states had a full ban on abortion (ie, AL, AR, ID, KY, LA, MS, MO, OK, SD, TN, TX, and WI), and 2 states had a gestational limit of 6 weeks (GA and OH) [13].

The reversal of abortion rights in the United States demands a ‘morality of caution’ around the collection of personal health information in health research that includes women and other people who can get pregnant [14]. The new political landscape surrounding abortion poses an immediate risk to participants, particularly those engaged in pregnancy or reproductive health-related research, but also those engaged in general biomedical research, where certain types of personal health information collected could be used to identify pregnancy termination events (eg, GPS data). This creates a pressing challenge for health researchers and a need to find solutions into the future. The objective of this viewpoint is to outline potential solutions for researchers that offer stronger protection for participants while maintaining integrity of research methods.

The obvious yet unfortunate solution for participants to completely reduce their personal risk and inadvertently offer any information that could be used against them is to not participate in health research. Further, participants in current research could request to have their personal health information deleted, akin to the European Union’s General Data Protection Regulation “Right to Be Forgotten” [15]; however, this relies on the participants having adequate knowledge of their risk as a participant, which is not always transparent, and the willingness of research institutions to honor such requests. We urge investigators and participants to consider alternative approaches so as to not impede progress in women’s health and reproductive research.

## Potential Solutions for Health Researchers

First, investigators should consider minimizing the information they collect, particularly sensitive information that could be used to indicate a miscarriage or pregnancy termination where this is not essential to meeting the study objectives. In these cases, researchers can consider discarding certain questions from existing surveys or questionnaires. If the data are not collected, they cannot be used to prosecute a participant. Although effective and able to preserve the data needed to meet the primary study objectives, this intervention omits data that could have added value in exploratory post hoc analyses or in integrated data sets. Therefore, in this approach, the consequences of censoring the collection of such data that are integral to health research should not be ignored.

There are two challenges to this aforementioned approach. First, open science research studies that collect reproductive health data that run without a data lock that controls access to research data—that is, data that are hosted in the public domain will be challenging to safeguard; in these open science environments, researchers may only be able to protect prospective participants through the removal of sensitive data fields before uploading the data. Second, if the information collected involves digital passive data, the removal of key sensitive fields becomes more complex. The increasing prevalence of digital health apps that are intended to provide useful information on the menstrual cycle and reproductive health, use a variety of smartphone-based tracking features, and often incorporate wearable devices poses potential risks to users. These risks are amplified by the substantial lack of regulation around the use of digital data from health-tracking apps and wearables [16], as such data are not subject to HIPAA regulations. Wearable devices capable of tracking physiological measures, such as body temperature and heart rate, have shown promise for tracking conception [17-20], and studies are starting to explore their potential for health monitoring in pregnancy [21]. These same signals could likely be used to identify miscarriage or termination signatures in study participants. This means that even without purposefully labeling miscarriage or termination events from surveys and questionnaires or health record data, a participant could still be at risk that their digital data be used to infer changes in their reproductive state or access to services. Additional passive data fields, such as GPS coordinates, activity, phone records, and many more, could also be used to determine abortion clinic access.

A second strategy for protecting research participant data from invasive investigation is for researchers to execute a strict and preferably automated anonymization protocol on any participant-level data as soon as a miscarriage or termination event occurs—that is, immediately deleting all personal identifiable data relevant to a participant who is no longer pregnant, including any keys linking study identifiers to personal identifiable data. In doing so, a substantial barrier between sensitive information and the participant is created. In light of the potential risk related to data acquired from wearable devices, this second alternative may be necessary to maximally protect participants. The consequence of this solution is that if it is executed during an active study period, those participants become untraceable and uncontactable for any follow-up study activities, resulting in inadvertent loss of other data points and potential study completion challenges. However, in the context of a research study where a pregnancy loss is a study end point, this consequence is likely to be less impactful. Additional consideration must be taken into ensuring this approach does not inadvertently further impose inequitable data deletions that differ by sex and gender. In using the aforementioned approaches, ensuring participants are fully informed prior to their participation and offering a choice is crucial.

Finally, efforts should be enhanced to develop reliable methods to generate synthetic data and other breakthrough technologies that preserve the value of the data while obfuscating the real data. Synthetic data sets are simulated data sets that retain the structure and statistical distribution of the original data set. When accurate, these artificially created data sets could be used in analysis and modeling without revealing the real-world data. However, outliers and small data sets remain challenging to simulate in a synthetic data set.

## Conclusions

As the reality of the Dobbs v. Jackson ruling sets in, we urge researchers to be proactive in activating processes and procedures to enable full engagement of women and others who can get pregnant in health research studies, while considering appropriate precautions for their privacy and safety now and in future studies. Although we have highlighted some solutions here, there are undoubtedly many other solutions that will surface as the political landscape continues to evolve. As a community, we must do everything possible to protect research participants, while also not impeding progress in reproductive and women’s health research.

## Abbreviations

HIPAA: Health Insurance Portability and Accountability Act
